# Biomechanics‐Driven 3D Architecture Inference from Histology Using CellSqueeze3D

**DOI:** 10.1002/advs.202518706

**Published:** 2025-12-05

**Authors:** Yan Kong, Hui Lu

**Affiliations:** ^1^ SJTU‐Yale Joint Center for Biostatistics and Data Science National Center for Translational Medicine Shanghai Jiao Tong University Shanghai 200240 China; ^2^ Department of Bioinformatics and Biostatistics School of Life Sciences and Biotechnology Shanghai Jiao Tong University Shanghai 200240 China; ^3^ Shanghai‐Chongqing Institute of Artificial Intelligence Chongqing 400000 China

**Keywords:** 3D Reconstruction, Biomechanical Constraints, Cell Squeezing, Computational Histology, Particle Swarm Optimization

## Abstract

Conventional 2D analysis of hematoxylin and eosin (H&E)‐stained images is fundamentally limited by the tissue thickness, as cellular overlap and morphological changes in the compressed perspective obscure distinct cell boundaries. To address this, it develops CellSqueeze3D, a computational framework that reconstructs the 3D spatial distribution and size of individual cells from a single H&E‐stained section. Founded on the principle that 2D cell compression preserves 3D geometry, the method employs a hybrid Particle Swarm Optimization (PSO) approach with biomechanical constraints to infer biologically plausible reconstructions. Validation shows that the nuclear‐to‐cytoplasmic (N/C) ratio distribution derived from the predicted cell radii differs significantly from random assignments (p = 1.39e‐80). By employing projected cell boundaries, the 3D‐informed cellular classifier surpassed traditional methods (AUC increases of 0.136 and 0.069). The resulting morphological metrics also revealed strong associations with key gene expression patterns, providing prognostic insights. Furthermore, cellular and nuclear size indices from CellSqueeze3D significantly predict the mutation status of 21 genes in TCGA cohorts, achieving a median AUROC above 0.65 in fivefold cross‐validation. This study demonstrates that fully utilizing the previously untapped 3D spatial information from a single slice significantly enhances computational pathology and quantitative tissue phenotyping.

## Graphical abstract

CellSqueeze3D reconstructs 3D cellular architecture from standard 2D histology images using biomechanical constraints and optimization. Validated on clinical datasets, it enables accurate tissue phenotyping, predicts gene mutations, and reveals significant correlations between nuclear‐cytoplasmic ratio entropy and tumor progression. This demonstrates the value of extracting 3D spatial information from routine pathology to advance cancer diagnostics.

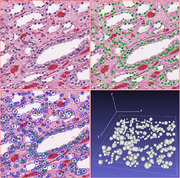

## 1 Introduction

Over the past decades, histopathology has undergone a profound transformation, driven by the integration of artificial intelligence (AI) and the rise of computational pathology.^[^ ^1^, ^2^ ^]^ These advancements have led to remarkable achievements in cancer prognosis prediction, tumor classification,^[^ ^3^ ^]^ and genetic mutation prediction.^[^ ^4^, ^5^, ^6^, ^7^ ^]^ More recently, 3D pathology has gained increasing attention as a promising frontier.^[^ ^8^, ^9^ ^]^ Studies involving serial sectioning and 3D reconstruction in cancers such as prostate^[^ ^10^ ^]^ and pancreatic carcinoma^[^ ^11^, ^12^ ^]^ have enabled in‐depth exploration of spatial tissue architecture, cellular distribution, and tumor heterogeneity.

While significant advances have been made in 2D‐to‐3D reconstruction across other imaging domains, for instance, breakthroughs in computed tomography (CT) have enabled the routine generation of 3D anatomical models from 2D X‐ray slices,^[^ ^13^ ^]^ and techniques in fluorescence microscopy now computationally reconstruct detailed 3D cell models from image stacks,^[^ ^14^ ^]^ the application to standard hematoxylin and eosin (H&E) slides still faces significant limitations. Methods based on serial sectioning are labor‐intensive, time‐consuming, and prone to image registration artifacts.^[^ ^15^, ^16^ ^]^ While advanced imaging techniques like confocal microscopy provide high‐fidelity 3D data, they are expensive, require specialized equipment, and are not widely accessible for routine clinical practice.^[^ ^17^ ^]^

Furthermore, a fundamental challenge persists in the analysis of individual 2D H&E slides. Standard tissue sections possess a non‐negligible thickness (typically 5–10 µm), meaning that cells are distributed at different depths along the z‐axis.^[^ ^18^, ^19^ ^]^ This inherent spatial information, though often overlooked, is critical for accurately interpreting tissue morphology. Existing computational methods that attempt to infer 3D structures from a single 2D slice often lack a robust framework for incorporating physical constraints. This deficiency leads to a cascade of negative consequences: without considering how cells physically interact, these models frequently produce biologically implausible reconstructions. For example, they may predict cell sizes that are inconsistent with their nuclei or place cells at positions that result in physical overlap or interpenetration. This fundamental flaw renders their outputs unreliable for downstream analysis and limits their utility for understanding true tissue biomechanics. The challenge is further compounded by inherent sampling and observational biases, where measured cross‐sectional cell radii are always smaller than their true 3D radii and smaller cells are underrepresented. While algorithms like EstiTiCS^[^ ^20^ ^]^ have been developed to correct these statistical biases and estimate overall 3D cell size distributions, they do not reconstruct the individual 3D position, size, and spatial relationships of each cell, which are critical for understanding tissue biomechanics.

We posit that the unique geometry of cell compression, a natural consequence of tissue preparation and evident in 2D H&E projections, offers a powerful and intrinsic constraint for 3D reconstruction. The phenomenon of “cell squeezing”, where adjacent spherical cells appear compressed but not interpenetrative in 2D, can be leveraged as a fundamental physical principle for spatial optimization. By combining this insight with other biological priors, such as the known range of the cell‐to‐nucleus radius ratio and the non‐overlapping nature of nuclei in 3D, we can formulate the problem as an inverse optimization task.

In this paper, we introduce CellSqueeze3D, a novel computational framework that infers the spatial 3D distribution and size of cells from a single H&E‐stained section. Our method leverages a hybrid optimization strategy that combines Particle Swarm Optimization (PSO)^[^ ^21^ ^]^ with biomechanical constraints, such as non‐interpenetration and cell‐to‐nucleus radius bounds, to ensure biologically plausible reconstructions. Our approach offers a number of significant advantages: compared to random distributions, our estimated cell sizes are more biologically meaningful; our cell‐centric image patches provide a more precise and accurate basis for cell‐type classification, overcoming the jagged‐edge artifacts seen in traditional small, square patches; and we have validated our method across multiple datasets. Our results demonstrate that our predicted nuclear‐to‐cytoplasmic (N/C) ratio correlates with key biological processes like cell division and can accurately predict both gene mutations and patient prognosis. By solving a complex inverse problem and extracting clinically meaningful information from standard pathology images, our work shows how classic optimization techniques, guided by physical principles, can offer a potential breakthrough in computational histology.

## 2 Results

### 2.1 PSO Training and Reconstructed Spatial Distribution

**Figure 1:**
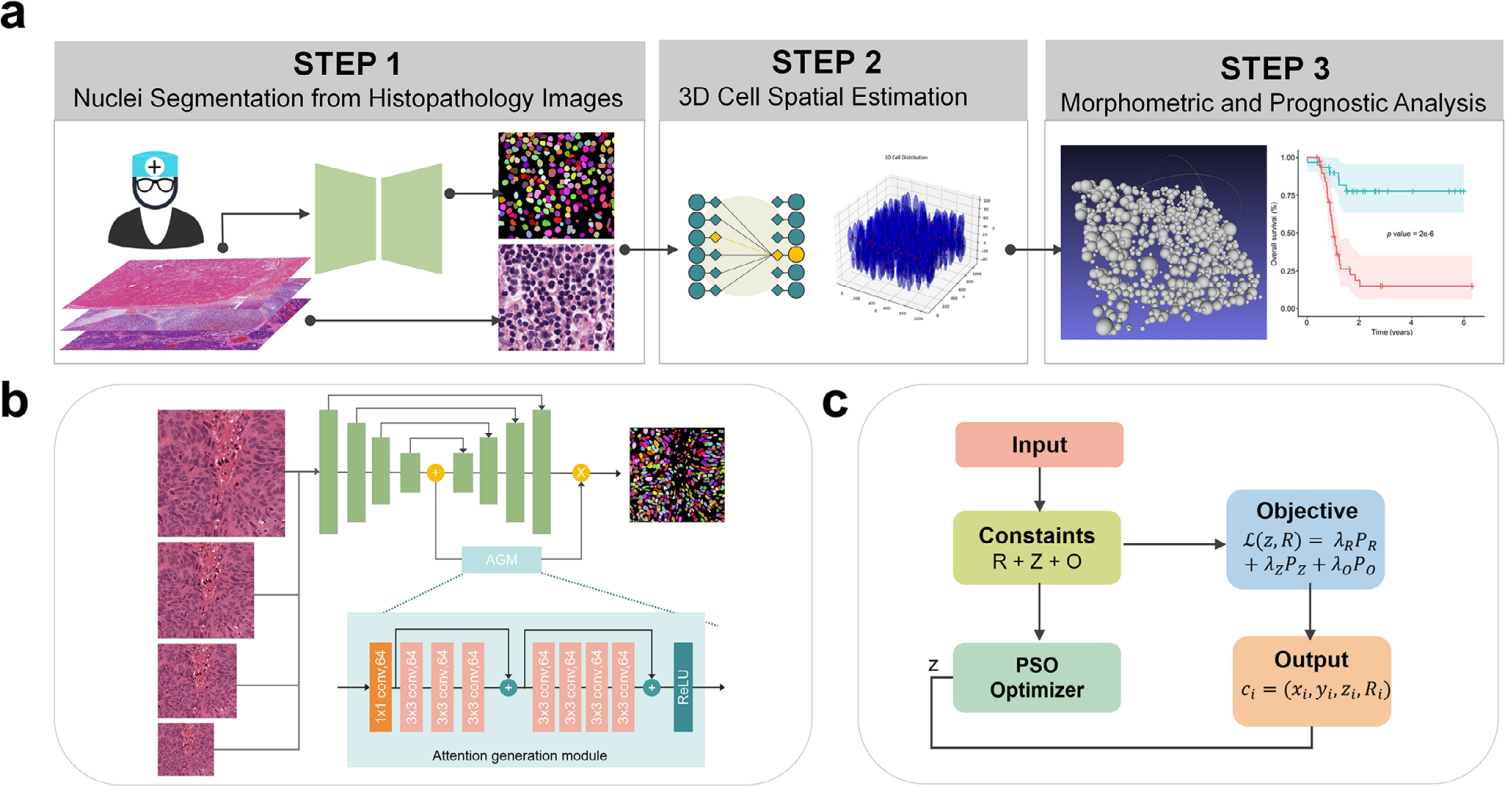
The CellSqueeze3D workflow for reconstructing 3D cellular architecture from a single H&E section. a) Overall pipeline. The process begins with instance‐level nuclear segmentation from an H&E image. The resulting nuclei are used as input for a Particle Swarm Optimization (PSO) model that infers 3D cell positions and sizes under biomechanical constraints. The output is a spatially reconstructed cell population enabling downstream analysis. b) Architecture of the multi‐scale nuclear segmentation model. c) Key steps of the PSO‐based optimization for reconstructing cell radius and z‐position.

**Figure 2:**
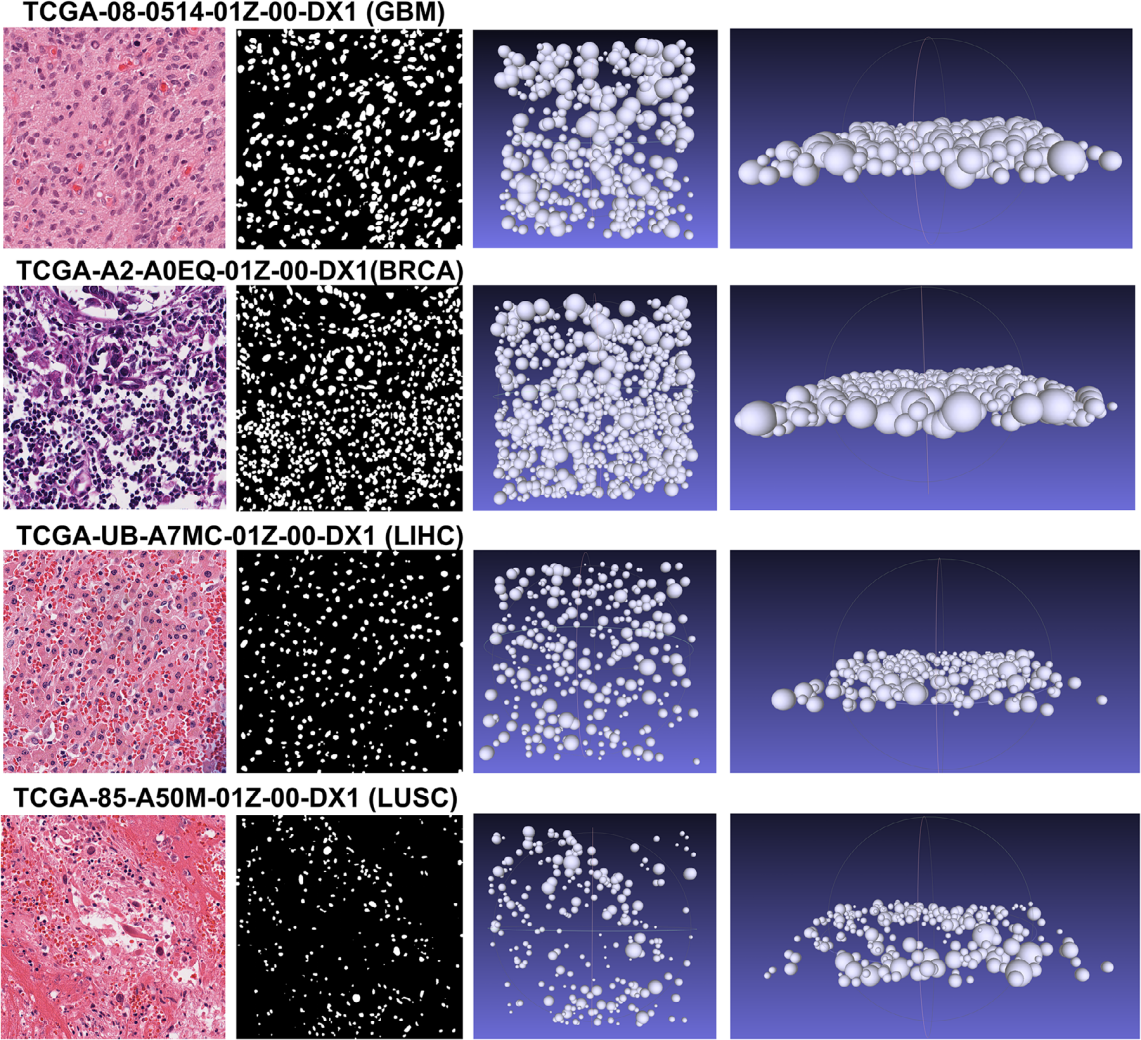
Representative examples of 3D cellular architecture reconstructed by CellSqueeze3D from H&E images in the TCGA cohort (sample barcodes indicated). From left to right: Original H&E image patch at 40x magnification; corresponding nuclear segmentation result; overhead (top‐down) view of the reconstructed 3D cell distribution, where spheres represent individual cells; side view of the 3D reconstruction, validating the inferred z‐axis distribution and non‐overlapping cell arrangement.

CellSqueeze3D can be trained efficiently on a single consumer‐grade machine. Each training session required ≈20 min (on a 4‐core CPU) for a 1000 × 1000 pixel patch image with 200 iterations and a particle swarm size of 50. The flowchart of the framework is shown in **Figure** **1**a. The predicted spatial distribution results are visualized in **Figure** **2**. The first column of Figure 2 displays a 40x magnification patch view of a representative HE image, the second column shows the nuclear segmentation results, the third illustrates the 3D reconstruction in a top‐down view, and the fourth column provides a side view, revealing the inferred Z‐axis distribution. These side views visually confirm that the algorithm successfully generates a non‐random, biologically plausible distribution of cells in the Z‐axis, with cells showing clear separation and minimal overlap. The program exports results in.PLY format, which can be imported into publicly available software such as MeshLab^[^ ^22^ ^]^ for rotation and smooth enlargement.

### 2.2 3D Coordinates Enhance Cell‐Type Classification

**Figure 3:**
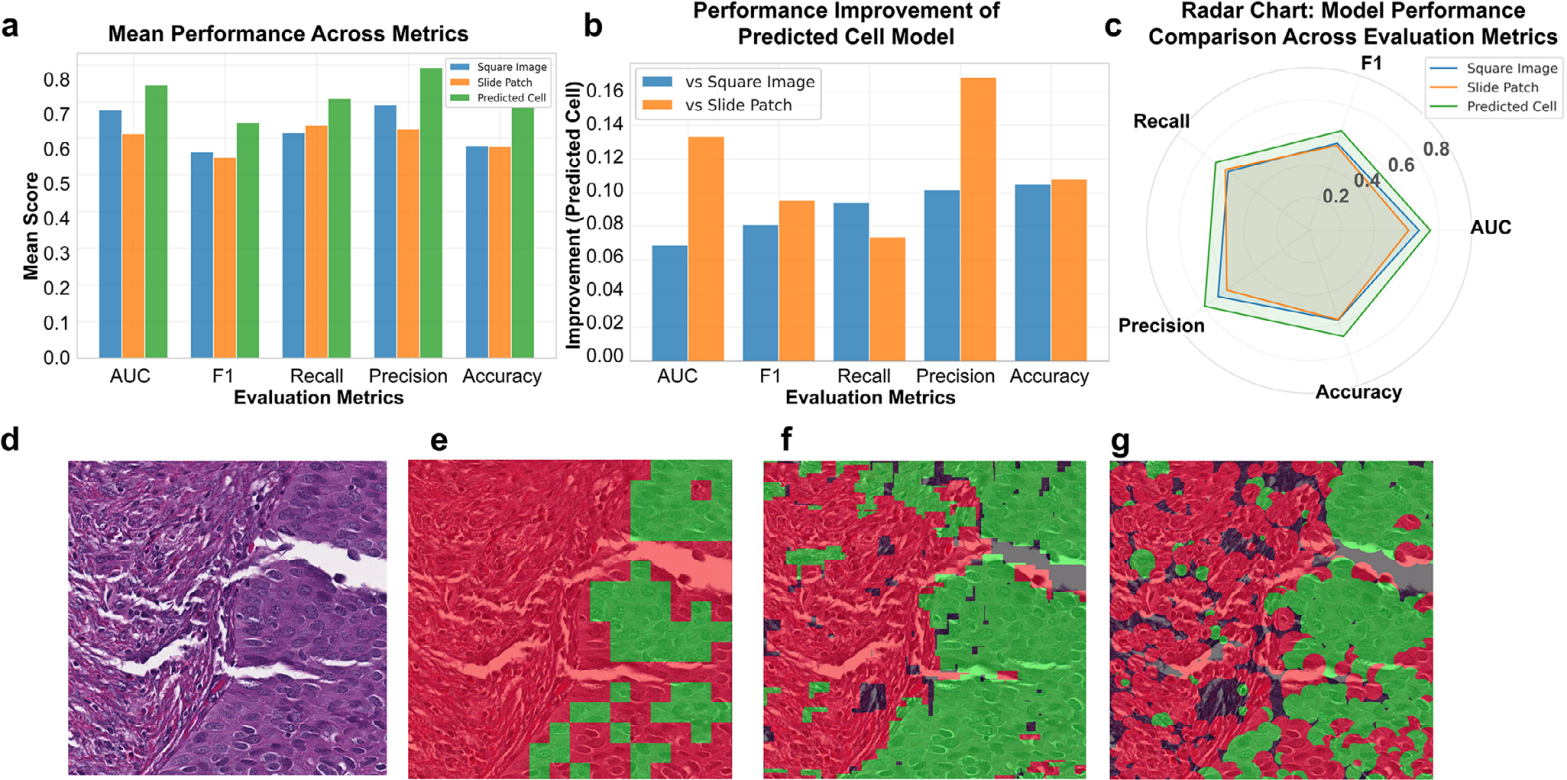
Comparative performance of tissue classifiers using 2D versus 3D‐informed cellular features. a) Summary of performance metrics across three datasets (total sample size, n = 1016). b) Average quantitative improvement in classification performance achieved by using 3D‐informed features over conventional 2D approaches, Performance was evaluated via fivefold cross‐validation, with 3D‐informed features yielding statistically significant enhancements (*p*<0.001, *t*‐test) across all five metrics compared to models using square image or slide patch inputs. c) Radar chart comparing five performance indices for a multi‐faceted evaluation. d–g) A representative example from the TCGA‐HNSC cohort (barcode: TCGA‐CV‐7242) illustrates the tumor region prediction results: d) Original H&E patch. e) Prediction from a model trained on small patches from a sliding‐window baseline. f) Prediction from a model using fixed square regions around nuclei (conventional 2D method). g) Prediction from our model using projected cellular regions derived from 3D reconstruction.

To directly quantify the benefit of our 3D reconstruction, we compared the performance of a classification model with identical architecture and training parameters across three different datasets. The results, as shown in **Figure** **3**a–c, demonstrate that the model trained on our predicted cellular regions consistently outperformed the models trained on the other two datasets across all five‐evaluation metrics. Specifically, compared to the common sliding‐window approach for constructing small patches, our method showed a significant improvement in precision of 0.164 and an AUC increase of 0.136. When compared to the nucleus‐centered square image method, our approach still improved precision by 0.105 and increased the AUC by 0.069. These findings are particularly noteworthy as the sliding‐window method is a widely used technique for tumor region classification. The superior performance of our approach indicates a significant gain in granularity and accuracy. Figure 3d–g provides a visual example of the prediction results from the different training sets on a representative test image.

### 2.3 Computational Validation on Synthetic Data

**Figure 4:**
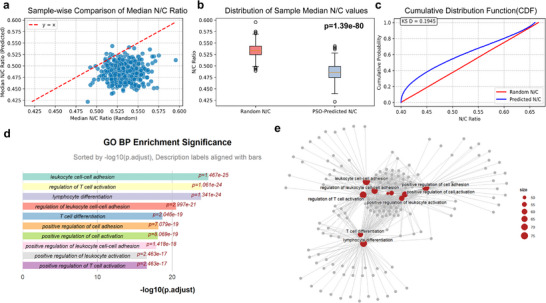
Statistical validation and biological correlation of the reconstructed cellular morphology. a–c) Statistical validation of the nuclear‐to‐cytoplasmic (N/C) ratio (sample size, n = 492; all patches are 1000 × 1000 pixels at 40X magnification): a) Scatter plot comparing N/C ratios derived from PSO‐optimized versus random cell sizes. b) Distribution of the median N/C ratio across different samples (*p*<0.001, Kolmogorov‐Smirnov test). c) Cumulative distribution function (CDF) plots confirming the significant difference (*p* < 0.05) between the optimized and random models. d,e) Biological relevance of N/C entropy: d) Top 10 enriched Gene Ontology (GO) Biological Process terms for genes correlated with N/C entropy. e) Network map showing the interconnections among the enriched GO terms.

We analyzed multiple samples across four TCGA datasets (Breast Invasive Carcinoma (BRCA), Lung Squamous Cell Carcinoma (LUSC), Kidney Chromophobe (KICH), and Lower Grade Glioma (LGG)), which included WSI slides and gene expression data. As shown in **Figure** **4**, which presents the computational comparisons for the BRCA dataset, we compared the entropy distribution of the nuclear‐to‐cytoplasmic (N/C) ratio produced by our algorithm against a null model distribution generated by random assignment of cellular sizes (Figure 4a,b). The entropy was quantified for each patch by first calculating the N/C ratio of all constituent cells and then computing the Shannon diversity index. The resulting distribution from our approach was significantly narrower and more biologically cohesive than that of the random model, as confirmed by a Kolmogorov–Smirnov (KS) test yielding *p* < 0.05 (Figure 4c). This indicates that our biomechanically constrained reconstruction captures more realistic and consistent morphological structures than stochastic assignments (Table S2, Supporting Information).

### 2.4 Functional Analysis of Genes Associated with N/C Entropy

In the BRCA dataset, we identified 875 genes significantly associated with N/C entropy (q‐value<0.05). N/C entropy is derived from the Shannon entropy applied to the distribution of the 3D‐informed Nuclear‐to‐Cytoplasmic (N/C) ratio across a defined tissue region. It serves as a quantitative measure of cellular morphological heterogeneity, reflecting the disorder or variability in cell size and nuclear size within the tumor microenvironment. Functional enrichment analysis revealed significant associations with biological processes critical for tissue architecture and cell behavior, such as cell‐cell adhesion, cell differentiation, and T cell activation (Figure 4d,e). This finding provides strong biological validation for our computational metric, linking it directly to the underlying genetic machinery that regulates tissue organization.

Similarly, we computed N/C entropy‐related genes for LUSC, LGG, and KICH samples. In LUSC, the genes were enriched in processes like mitotic cell cycle phase transition and cell differentiation, processes known to be dysregulated in lung squamous cell carcinoma (Figure S1, Supporting Information). In LGG, the genes were associated with mitotic nuclear division, chromosome segregation, and organelle fission‐ biological processes directly related to uncontrolled cell growth characteristic of gliomas (Figure S2, Supporting Information). For KICH, the significant genes were enriched in processes related to nuclear division, mitotic nuclear division, and sister chromatid segregation, highlighting the relevance of our morphological metric to cell proliferation in kidney cancer (Figure S3, Supporting Information). These cross‐dataset findings underscore the generalizability of our approach and its ability to capture fundamental biological signals from morphological data.

### 2.5 Patient Prognosis Prediction

**Figure 5:**
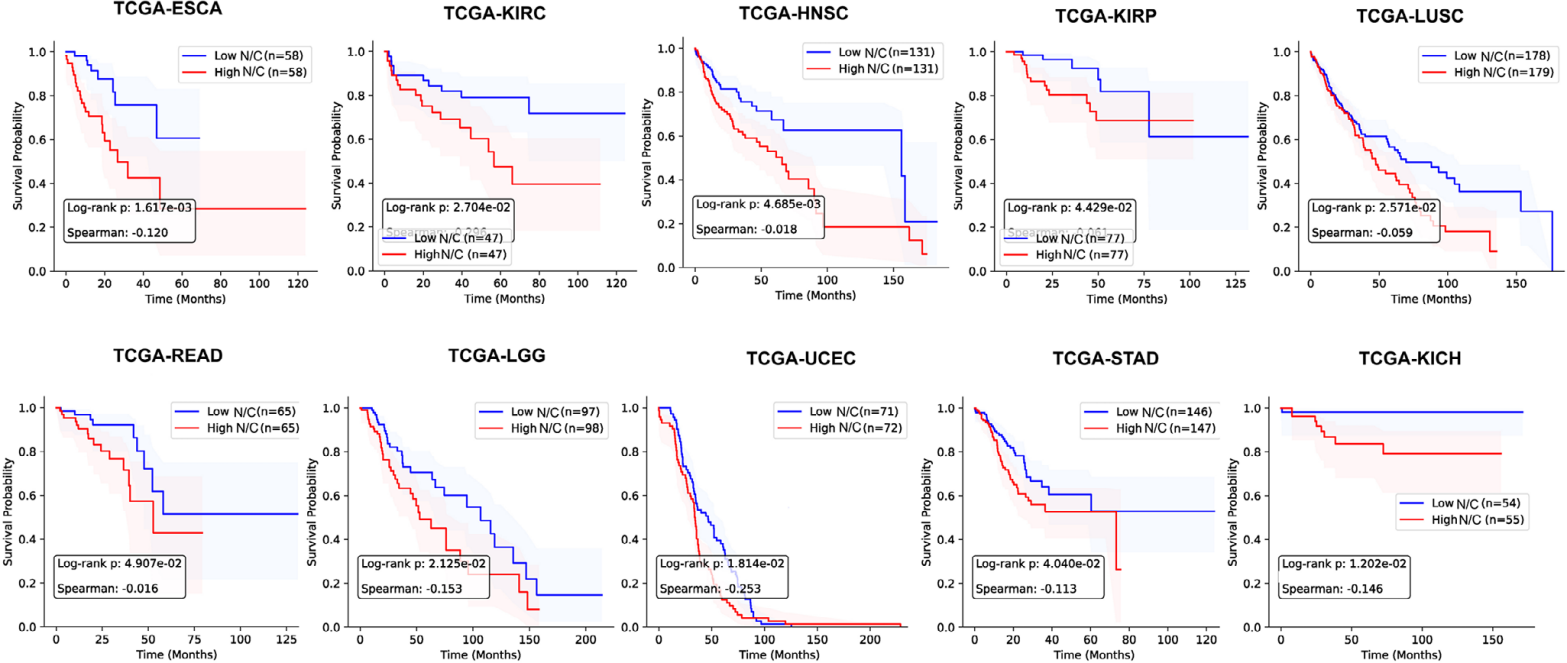
Kaplan‐Meier survival curves based on the N/C ratio for patient prognosis. The figure demonstrates a statistically significant separation between high and low N/C entropy patient groups.

In 10 of the cancer types analyzed, N/C entropy significantly distinguished between high‐risk and low‐risk patients, as demonstrated by Kaplan‐Meier survival curves (**Figure** **5**). Patients with higher N/C entropy values exhibited significantly worse overall survival compared to those with lower values. Log‐rank test p‐values for these curves were all below 0.05, and Spearman correlation coefficients were consistently negative. This counterintuitive finding – suggesting that greater heterogeneity may be associated with poorer outcomes‐ challenges prior hypotheses and implies that the specific morphological variations captured by our model, potentially reflecting disordered and uncontrolled growth, may signify more aggressive disease behavior. These results underscore the potential value of N/C entropy as a prognostic biomarker in clinical oncology.

### 2.6 Gene Mutation Prediction and Spatial Analysis

**Figure 6:**
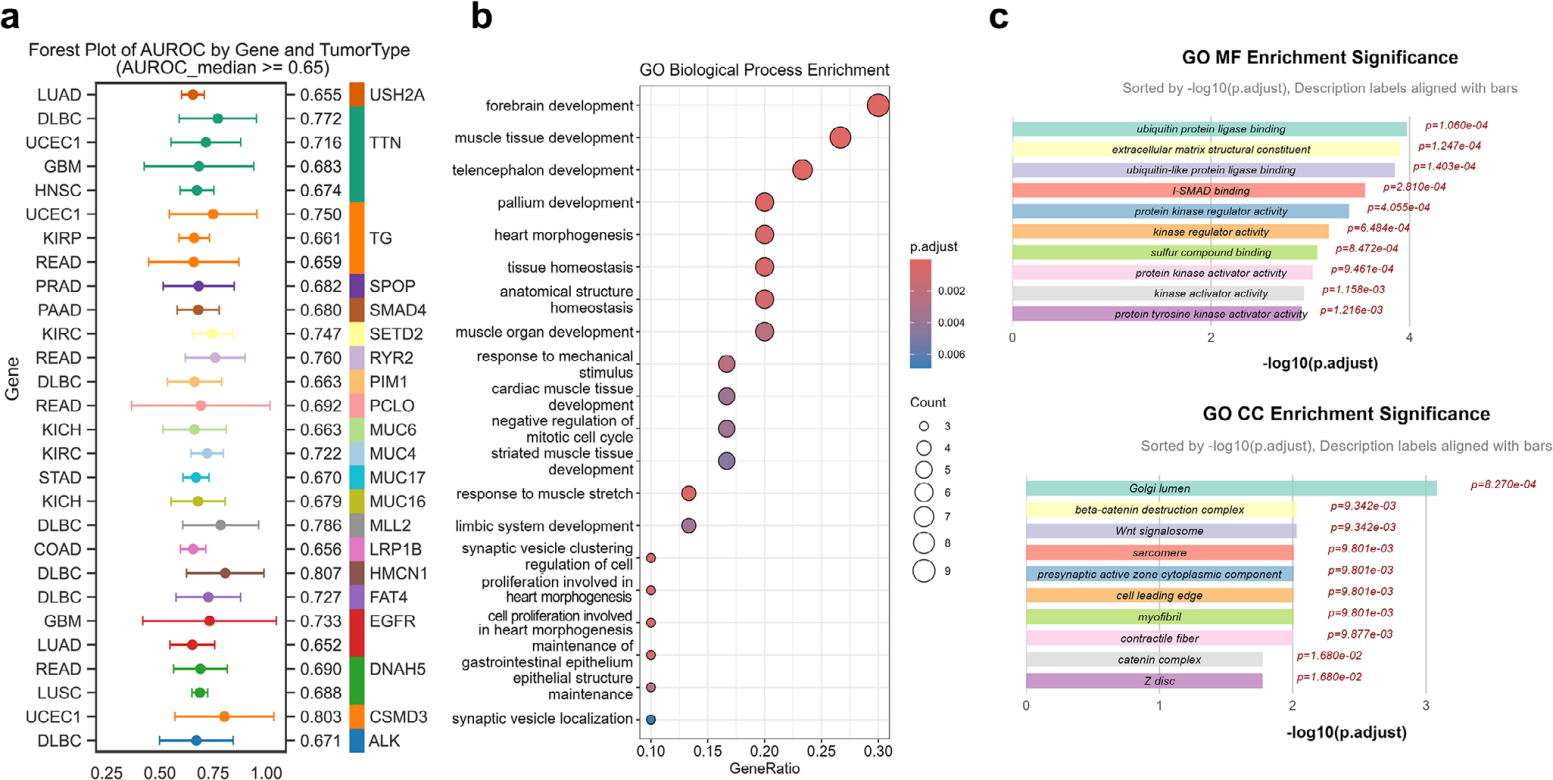
Predictive power of the N/C ratio for gene mutations and their functional relevance. a) Forest plot of the Area Under the Receiver Operating Characteristic Curve (AUROC) for predicting gene mutations across tumor types. The analysis encompassed 36 tumor types with a total sample size of 5453. Displayed are genes with a median AUROC > 0.65 from fivefold cross‐validation. b,c) Functional enrichment analysis of the predictable genes: b) Top enriched Gene Ontology (GO) Biological Process terms. c) Enriched GO terms for Cellular Component and Molecular Function, providing insights into subcellular localization and molecular activities.

Using the N/C entropy values, we built gene mutation prediction models for 23 cancer types. In a fivefold cross‐validation, we identified 21 genes that could be effectively predicted using N/C entropy, with a median AUROC greater than 0.65. This result indicates that our derived morphological metric contains predictive power for specific genetic alterations. **Figure** **6**a presents a forest plot of the AUROC for these predictions. Functional analysis of the 21 genes revealed significant associations with multiple organ development and tissue homeostasis processes, which are often disrupted in cancer (Figure 6b).

## 3 Discussion

### 3.1 Briding the 2D to 3D Gap in Histology

CellSqueeze3D represents a significant leap in computational histology, providing the first algorithmic solution to the “2D to 3D inverse problem” that is grounded in biomechanical principles. Unlike previous approaches that rely on complex imaging technologies or idealized simplistic assumptions, our method leverages the inherent biomechanical constraints encoded in a standard 2D histology image. By modeling cells as a system of independent, interacting spheres subject to the biochemical constraint of non‐interpenetration (compression without overlap), our approach shifts from mere inference to constrained optimization, thereby producing biologically realistic results. This innovative framework paves the way for accurate and cost‐effective 3D cell architecture modeling in clinical pathology, democratizing access to crucial spatial information.

#### 3.1.1 Comparison with Deep Learning Depth Estimation

Deep learning (DL) models applied to computer vision for depth estimation typically treat 3D reconstruction as a regression problem. They require paired data where 2D images are the input, and the true 3D coordinates or depth map (often obtained from serial sectioning or confocal microscopy) serve as the ground truth labels for training.^[^ ^23^, ^24^, ^25^ ^]^

The primary limitation of these DL models is their complete dependence on the quality and volume of the labeled 3D ground truth data. In the field of histology, the generalizability of these models is restricted because meticulously labeled 3D histological ground truth is extremely scarce, expensive to acquire, and often prone to sectioning artifacts and registration errors. Furthermore, DL models are generally “black boxes”; they learn correlations but do not guarantee that the predicted depth adheres to fundamental physical or biological principles (e.g., that cells cannot interpenetrate).

In contrast, CellSqueeze3D solves the 3D structure problem through biomechanical optimization based on the principle of minimal potential energy (non‐interpenetration). It is data‐independent of 3D ground truth, relying solely on the geometry extracted from a single 2D H&E image (nuclear boundaries and size). This makes our results inherently biologically plausible and highly interpretable because they satisfy explicit physical constraints at every step of the reconstruction.

#### 3.1.2 Comparison with Advanced Segmentation Models (SAM Variants)

Foundation segmentation models like SAM2 or SAM3 focus on generating highly accurate 2D masks based on prompts or representations. These models are optimized for 2D instance or semantic segmentation (delineating cell nuclei, tissue boundaries, etc.). While they excel at defining the nuclear boundaries (the first step input for CellSqueeze3D), they provide no 3D information (Z‐depth or cell size). These advanced DL segmentation tools are complementary to CellSqueeze3D; they can be used to generate superior quality and highly robust nuclear masks, which would serve as a high‐quality input layer for our optimization framework. They do not, however, offer a solution to the fundamental inverse problem of inferring cellular size or 3D spatial distribution.

However, recent advancements include models like meta SAM 3D, designed for general‐purpose 3D object and scene reconstruction from natural images. While this marks a significant step toward common sense 3D understanding in computer vision, its application context remains distinct from ours. SAM 3D is trained on vast datasets of natural, real‐world images (such as human bodies, animals, and objects) and corresponding 3D meshes. Its goal is visually grounded reconstruction of macroscopic objects that have defined shapes and textures. This technology is not directly applicable to histology images because cellular architecture involves microscale, soft‐body interactions and lacks the distinct, rigid textures of macroscopic objects. Furthermore, training SAM 3D for cells would require a histology‐specific 3D ground truth dataset, which, as we previously discussed, is extremely scarce and prone to sectioning artifacts.

Therefore, whether using the 2D segmentation models (SAM2, SAM3) or the general 3D reconstruction model (SAM 3D), these methods remain complementary to CellSqueeze3D. They can generate high quality and robust nuclear masks (input), but they do not solve the fundamental inverse problem of inferring cellular size and 3D spatial distribution based on biomechanical constraints, which is a problem unique to tissue packing and histology analysis. CellSqueeze3D provides the specialized, physics‐informed engine required for accurate cellular‐level 3D morphology in pathology.

CellSqueeze3D's contribution lies in its hybrid, physics‐informed approach. It tackles the core challenge, inferring physically constrained 3D distribution and cellular size, without relying on the scarce 3D ground truth required by DL regression methods. Instead of being a purely data‐driven model, CellSqueeze3D serves as a robust, interpretable engine for generating physically realistic 3D architectural features, which can then be used to create the high‐dimensional features (as shown by our AUC and AUROC results) that subsequent DL classifiers (GNNs, Transformers, etc.) can learn from.

### 3.2 3D Morphological Features in Multi‐Omics and Functional Prediction

While the core contribution of CellSqueeze3D lies in solving the 2D‐to‐3D inverse problem, its ultimate value is providing foundational features for advanced pathological functional analysis. Traditional 2D morphological features have already been demonstrated to correlate with gene expression and molecular signatures.^[^ ^26^, ^27^ ^]^ Building upon this, the 3D cellular architecture features derived by CellSqueeze3D offer a more accurate bridge connecting morphology with functional omics.

### 3.3 Clinical and Computational Impact

This method holds immense potential to transform clinical pathology. The link between cellular morphology and disease prognosis isn't a new concept. Early studies in multiple cancers have long shown that morphological features can provide insights into patient outcomes.^[^ ^28^ ^]^ Our method takes this a significant step further. By providing pathologists with a reliable and accessible way to visualize and quantify 3D cellular architectures, our approach can inform diagnosis, guide treatment planning, and improve prognostic accuracy. The ability of CellSqueeze3D to derive the N/C ratio and link it to patient prognosis and gene mutations demonstrates that simple morphological metrics, when derived from a biologically informed model, can serve as powerful biomarkers. This offers a new layer of quantitative analysis that complements traditional visual assessment, providing a more nuanced understanding of cellular morphology and its role in disease.

Furthermore, our results in cell‐type classification highlight a key advantage of our approach over traditional methods. Common tumor region classification models often use a sliding‐window approach. While effective, such method generates numerous small patches, of which contain unnecessary non‐cellular regions, thereby increasing computational overhead. Moreover, increasing the patch size to capture larger contextual information often results in a loss of fine‐grained boundary detail. Our method overcomes these limitations by constructing cell‐centric patches based on the predicted 3D cellular boundaries. The significant improvement in accuracy and precision (0.164 and 0.136 respectively) over the sliding‐window method proves that our approach can achieve superior performance at a finer, more granular level. By focusing on biologically meaningful units rather than arbitrary grid‐based patches, we enhance both the precision and interpretability of our classification. This offers a new layer of quantitative analysis that complements traditional visual assessment, providing a more nuanced understanding of cellular morphology and its role in disease.

### 3.4 Limitations and Future Directions

While a significant step forward, our approach has certain limitations, particularly concerning the diversity of tissue architecture. Addressing these context‐dependent challenges will be crucial for extending CellSqueeze3D's applicability across the entire spectrum of pathological tissues.

1. Assumption of Cell Geometry (Tissue Type Dependence): The primary assumption of a spherical cell shape is a reasonable and effective simplification for many epithelial and immune cell types, especially in the context of tumor tissues which often exhibit increased nuclear circularity. However, this assumption may not be applicable to tissues characterized by highly anisometric or irregular cellular morphologies, such as those found in muscle or nervous tissue.^[^ ^29^, ^30^ ^]^ For instance, cells in muscle tissue (such as long, spindle‐shaped smooth muscle cells), nervous tissue (such as highly branched neurons and glial cells), or fibrous connective tissue (such as elongated fibroblasts) exhibit complex non‐spherical geometries. In these contexts, modeling them as spheres would likely introduce substantial reconstruction error. Future work will need to explore more complex cell geometries, such as ellipsoids or generalized polyhedra, to accommodate this biological variability.
2. Handling of Extreme Density and Tissue Architecture: The optimization problem becomes increasingly complex in regions of extremely high cell density (such as high‐grade lymphoma or extremely packed epithelial tumors). In such areas, there may be inherent ambiguities in finding a unique, optimal solution.^[^ ^29^, ^30^ ^]^ Furthermore, the current framework may face challenges in distinguishing between true cellular crowding^[^ ^31^ ^]^ and artifacts from the tissue sectioning process.^[^ ^32^ ^]^ This ambiguity is compounded in tissues like lymphoid organs where cells naturally exhibit minimal cytoplasm and maximum packing efficiency.
3. Tissue Heterogeneity and Artifacts: The model currently relies on local interactions and biomechanical constraints. It does not account for other complex biomechanical factors, such as cell‐to‐cell adhesion cell‐to‐cell adhesion,^[^ ^33^, ^34^ ^]^ cytoskeletal tension,^[^ ^35^ ^]^ or extracellular matrix interactions,^[^ ^36^ ^]^ into the objective function, nor does it model the extracellular matrix (ECM). The ECM can significantly influence cell shape and distribution in tissues like desmoplastic tumors. Furthermore, processing artifacts, such as folds, tears, or uneven staining, can disproportionately affect the local density and morphological features derived from the 2D projection, potentially leading to errors in the inferred 3D reconstruction in those specific regions.
4. Applicability to Non‐Spherical Nuclei: While H&E staining clearly delineates nuclear boundaries, the model currently prioritizes inferring the spherical cell body based on the nuclear center. In tissues where nuclei themselves are highly anisometric or lobulated (such as inflammatory cells like neutrophils or certain types of sarcomas), the use of a simple 2D nuclear centroid as the starting point for a spherical cell may not be optimal.

### 3.5 Future Enhancements

Our work is deeply rooted in the theoretical framework of epithelial packing, which posits that cell arrangements are optimized to minimize energy and maximize space efficiency.^[^ ^37^, ^38^, ^39^, ^40^ ^]^ This foundational principle can be further extended. Future research could incorporate other complex biomechanical forces, such as cell‐to‐cell adhesion, cytoskeletal tension or extracellular matrix interactions integrating these forces could lead to even more accurate and biologically faithful 3D reconstructions. Future work will need to explore more complex cell geometries, such as ellipsoids or generalized polyhedra, to accommodate the biological variability found in muscle and nervous tissues. The combination of our optimization‐based framework with modern deep learning techniques, such as Graph Neural Networks^[^ ^41^ ^]^ and transformer,^[^ ^42^ ^]^ holds significant potential to enhance both scalability and accuracy by learning complex, high‐dimensional representations of tissue structure. We note that recent related work has begun leveraging GNNs to model complex cellular systems and mechanical interactions,^[^ ^43^, ^44^, ^45^ ^]^ confirming the feasibility of this integration. Specifically, GNNs could be utilized to learn the actual cell‐to‐cell interaction forces to refine our optimization objective function. Alternatively, GNNs could predict the ideal 3D topological structure, providing this spatial arrangement as a hard constraint to guide the optimization process. This structure‐guided approach would mitigate the risk of converging to biologically implausible local minima in high‐density regions. Integrating these forces and leveraging the predictive power of deep learning will lead to even more accurate and biologically faithful 3D reconstructions. This combined approach would allow us to go beyond simple morphological metrics and learn the subtle, high‐dimensional features of disease with unprecedented accuracy.

## 4 Conclusion

The proposed CellSqueeze3D introduces a novel paradigm in computational histology, offering a unique and powerful solution to the long‐standing challenge of 3D cellular architecture reconstruction from single histological sections. By leveraging a biomechanical constraint‐guided optimization approach, we have shown that it is possible to extract biologically and clinically relevant 3D information from standard 2D pathology images without relying on scarce 3D ground truth data required by typical deep learning methods. Our findings demonstrate that our derived morphological metrics, such as the refined N/C ratio distribution, are not random artifacts but are significantly correlated with fundamental biological processes, gene expression, and patient outcomes. The superior performance of our method, particularly in tumor region classification, validates our focus on the individual cell as the fundamental unit of analysis and highlights a significant advantage over conventional 2D methods.

Looking ahead, we envision a future where our method seamlessly integrates with deep learning techniques, such as Graph Neural Networks (GNNs) and transformers. This hybrid approach will enable the reconstruction of complex, 3D structures and allow us to learn subtle, high‐dimensional features of disease structure with unprecedented accuracy, moving beyond simple morphological metrics. This integration holds potential for more refined 3D reconstruction of entire tissues, offering powerful new models for studying tumor heterogeneity, visualizing specific tumor microenvironment characteristics, and even predicting tumor evolution. Furthermore, the structured and physically constrained 3D morphological features provided by CellSqueeze3D will become an important input for the next generation of automated scientific discovery models. For instance, automated computational paradigms aim to automatically conceive scientific problems, establish models, run, debug, and iteratively search for solutions.^[^ ^46^, ^47^ ^]^ Our 3D feature collection can serve as high‐quality, interpretable prior knowledge for these automated systems, accelerating the generation and validation of new biological hypotheses in pathology, thereby driving revolutionary advancements in the study of tumor heterogeneity and drug target discovery.

This work lays the foundation for a burgeoning field of “Computational Histology,” where the joint power of physics‐based modeling and artificial intelligence unlocks a new era of quantitative pathology.

## 5 Experimental Section

### Datasets

The computational framework relies on a meticulous data preparation pipeline to transform raw histological images into structured input for the optimization model. The workflow is as follows:

1. Whole Slide Image (WSI) Acquisition: The utilized Formalin‐Fixed‐Embedded (FFPE) Hematoxylin and Eosin (H&E) stained tissue section WSIs from The Cancer Genome Atlas (TCGA) database^[^ ^48^ ^]^ and downloaded the .svs format files. These gigapixel images serve as the foundation for the analysis. After excluding WSIs with missing baseline resolution end up with 9779 WSIs (Table S1, Supporting Information). it used 24 cancer sub‐types spanning form 34 anatomic tissue sites (based on ICD‐0 code).

Tumor Region Annotation: Expert pathologists manually annotated the tumor boundaries on the pathological images, providing the ground truth for tumor regions.

1. Patch Extraction: Using the OpenSlide Python library, it extracted representative high‐magnification fields of view (40X) from the WSIs. These fields were cropped into smaller image patches of a fixed size (e.g., 1000×1000 pixels) and saved as standard image files (e.g., PNG or TIFF). This process focused the analysis on regions of interest while making the data computationally manageable.
2. Nuclear Segmentation: For each extracted HE image patch, a previously validated, pre‐trained nucleus segmentation model was used to identify individual cell nuclei.^[^ ^49^ ^]^ This model provided pixel‐level masks for each nucleus.
3. Nuclei Instance Calculation: From the segmentation results, the essential input variables were calculated for the optimization model: the 2D centroid coordinates (x_i_, y_i_) and the nuclear radius R_i_ for each detected nucleus using watershed Algorithm. These features, along with the original HE image patch, constituted the final input data for the CellSqueeze3D framework.

To obtain patient point mutation statuses, the downloaded mutation data from cBioportal.^[^ ^50^ ^]^ After excluding WSIs with missing baseline resolution, 69 genes were obtained in 5453 slides (Table S1, Supporting Information).

In survival analysis, Esophageal squamous cell carcinoma (ESCA, n = 116) was used, Renal clear cell carcinoma (KIRC, n = 94), Head and neck squamous cell carcinoma (HNSC, n = 266), Renal papillary carcinoma (KIRP, n = 154), Lung squamous cell carcinoma (LUSC, n = 357), Rectal cancer (READ, n = 130), Low‐grade glioma (LGG, n = 195), Endometrial cancer (UCEC, n = 143), STAD(293), and Renal cortical cell tumor (KICH, n = 109). The overall survival (OS) information was collected from the TCGA website. Patients with missing OS data were used as censored data in this study.

In the correlation analysis between gene expression and the nuclear/cellular ratio, first the overlapping samples were identified between the gene expression matrix and the whole‐slide image (WSI) dataset. For each matched sample, five image patches were randomly selected for subsequent computation. The analysis incorporated samples from breast cancer (n = 492, patch average cell number = 137), lung squamous cell carcinoma (n = 416, patch average cell number = 944), and low‐grade glioma(n = 212, patch average cell number = 499). The gene expression matrix was obtained from the TCGA database, and log‐transformed normalized counts were used as quantitative measures of gene expression.

### Mathematical Formulation of the Spatial Cell Distribution Model

A computational framework was designed to infer the pseudo‐3D distribution of cells from a single 2D hematoxylin and eosin (H&E) stained image, in which only nuclear positions and radii are available. The overall workflow of the framework is summarized in Figure 1a, which outlines the main pipeline from nuclear segmentation to 3D reconstruction, presents the architecture of the multi‐scale segmentation model (Figure 1b), and details the key steps of the PSO‐based optimization for reconstructing cellular architecture (Figure 1c). The key of the model is to determine the optimal Z‐coordinates and full cell radii that satisfy a set of biomechanical constraints.

### Input and Variables

For each detected nucleus *i*∈{1,…,N}, the input consists of the 2D centroid coordinated (*x_i_*, *y_i_*) and the nuclear radius $r_{i}^{\mathrm{nuc}}$. The optimization variables are the cell z‐coordinates: *z*  =  (*z*_1_, …,  *z_N_*) and whole cell radii: *R*  =  (*R*_1_, …,  *R_N_*). The 3D center of each cell is then defined as *c_i_* =  (*x_i_*, *y_i_*, *z_i_*).

### Initialization

The model parameters will be initialized based on the radius of the cell, and they will vary within a fixed range, defines as: $z_{i}^{\left(0\right)}∼U(0,\;z_{\max}),\;R_{i}^{\left(0\right)}∼U(\alpha \;\cdot \;r_{i}^{\mathrm{nuc}},\beta \cdot \;r_{i}^{\mathrm{nuc}})$. The range limit α and β need to be set according to different types of tumors, and set α = 1.5 and β = 2.5 as the default value.

When the distance between two nuclei in the 2D plane is less than the sum of their radii, their Z‐coordinates are randomly adjusted to prevent spatial overlap and ensure a valid initial configuration. **Table** **1** lists the key parameters used in the CellSqueeze3D algorithm, along with their explanations and default values.

**Table 1:** Parameter Settings for CellSqueeze3D.

Symbol	Meaning	Default value
Α	Minimum ratio of cell/nucleus radius	1.5
Β	Maximum ratio of cell/nucleus radius	2.5
z_max_	Maximum depth range	40–50 (µm)
λ_R_​	Penalty for radius violation	5 × 10^4^
λ_Z_	Penalty for z‐axis violation	1 × 10^4^
λ_O_	Penalty for overlap	1 × 10^6^
w_start_, w_end_	Inertia weight schedule	0.9–0.4
c_1_,c_2_	Cognitive/social coefficients	2.5–1.0, 1.0–2.6
n_swarm_	Swarm size	50
T	Iterations	100–120

### Penalty Functions

To enforce the biomechanical constraints, it introduces a series of penalty terms that are minimized during the optimization process:

1. **Radius Bounds**: This term penalizes cell radii that fall outside the biologically plausible range relative to their corresponding nuclear radius, ensuring that the reconstructed cell size is realistic. The penalty is formulated as:
(1) $$P_{R}=\sum_{i=1}^{N}\max(0,\;\alpha \cdot \;r_{i}^{\mathrm{nuc}}-R_{i})+\sum_{i\;=1}^{N}\max(0,R_{i}-\beta \cdot \;r_{i}^{\mathrm{nuc}})$$
2. **Z‐axis Bounds**: The depth of the cells (Z‐axis coordinates) is constrained within predefined limits, typically the thickness of the histological section, to prevent physically impossible reconstructions. The penalty is given by:
(2) $$P_{Z}=\sum_{i=1}^{N}\max(0,\;z_{min}-\;z_{i})+\max(0,\;z_{i}-z_{\max})$$
3. **Cell Overlap (3D minimal distance enforced by nuclear radii)**: This is the most critical biomechanical constraint. We enforce a minimal distance between adjacent cells in 3D space to prevent physical interpenetration. The neighbor set N_i_ for each cell i is defined via KD‐Tree search with a proximity threshold, ensuring that we only consider adjacent cells. The penalty function is then:
(3) $$P_{O}=\sum_{i=1}^{N}\sum_{j\in N_{i}}\max(0,(r_{i}^{\mathrm{nuc}}+r_{j}^{\mathrm{nuc}})-∥c_{i}-c_{j}∥)\;$$

Here *N_i_* denotes the neighbor set obtained via KD‐Tree search with threshold τ =  2maxR_*i*_, ‖c_*i*_‐c_*j*_‖ is the Euclidean distance between the 3D centers of cells *i* and *j*.

### Objective Function

The overall loss function is defined as the weighted sum of the penalty terms, aiming to find a solution that minimizes all constraint violations simultaneously:

(4) $$L(z,R)=\lambda_{R}\;P_{R}+\lambda_{Z}\;P_{Z}+\lambda_{O}\;P_{O\;}$$

The weights (λ_*R*_, λ_*Z*_, λ_*O*_) are determined empirically to balance the influence of each constraint.

### Optimization with Particle Swarm Optimization (PSO)

A hybrid optimization approach combining Particle Swarm Optimization (PSO) and a genetic algorithm was utilized for its ability to efficiently explore complex, non‐linear search spaces. Each particle in the swarm encodes a potential solution with the cell parameters: x  =  (*z*_1_,…,  *z_N_*, *R*_1_,…,  *R_N_*) ∈ R^2*N*^. At iteration *t*, the velocity and position of each particle are updated as:

(5) $$v_{k}^{t+1}=w^{\left(t\right)}v_{k}^{t}+c_{1}^{\left(t\right)}r_{1,k}⊙\left(p_{\mathrm{bes}t_{k}}-x_{k}^{t}\right)+c_{2}^{\left(t\right)}r_{2,k}⊙\left(g_{\mathrm{bes}t_{k}}-x_{k}^{t}\right)$$

(6) $$x_{k}^{t+1}=\Pi_{B}{\;(x}_{k}^{t}{+v}_{k}^{t+1})$$

where $r_{1,k},r_{2,k}∼U\left(0,1\right),⊙$ denotes element‐wise multiplication, and Π_B_ projects onto the feasible domain, B, ${\;x}_{k}^{t}$ and $v_{k}^{t}$ are the position and velocity vectors of the particle, respectively, $p_{\mathrm{bes}t_{k}}$ is the best position found by the individual particle, and $g_{\mathrm{bes}t_{k}}$ is the best position found by the entire swarm. The inertia *w* ^(*t*)^ decays linearly from a high value (e.g., 0.9) to a low value (e.g., 0.4) to balance exploration and exploitation, with c_1_ decreasing and c_2_ increasing over time to shift the focus from individual search to global convergence.

### Output Representation

The reconstructed cells are modeled as spheres, defined by their 3D centers (*x_i_*, *y_i_*, *z_i_*) and radii R_*i*_. The reconstructed cells are modeled as spheres:

(7) $$S_{i}=\left\{\left(x,y,z\right)|{\left(x-x_{i}\right)}^{2}+{\left(y-y_{i}\right)}^{2}+{\left(z-z_{i}\right)}^{2}=R_{i}^{2}\right\}$$

and the final 3D coordinates can be exported to standard 3D meth formats (e.g., PLY) for visualization and subsequent quantitative analysis.

### Synthetic Validation

To validate the optimization process, the reconstructed cell radii and Z‐coordinates were compared against two baselines: 1) a distribution of randomly generated values (from a range of 1.5 to 2.5 times the nuclear radius which equals to default limits), and 2) a “naive” uniform distribution. The entropy of the nucleus‐to‐cytoplasm (N/C) ratio distribution was calculated for both the optimized and random predictions. The Kolmogorov‐Smirnov (KS) test was used to assess the statistical significance of the differences between the optimized values and the random predictions.

To further evaluate the clinical relevance of the morphological metrics, the entropy of the cell area and predicted cell volume were calculated for each sample. Then the distribution of these entropy values was analyzed across different TNM staging categories (Tumor, Node, Metastasis) and performed statistical significance tests to determine if there were meaningful differences between stages.

Furthermore, the correlation was examined between the N/C ratio and gene expression data from multiple datasets (BRCA, LUSC, LGG, and KICH) obtained from The Cancer Genome Atlas (TCGA) database. Genes were identified that significantly correlated with the Nucleus/Cell radius ratio (N/C, *q*‐value < 0.05) and subjected them to functional enrichment analysis using hypergeometric tests.

### Cell‐Type Classification

To evaluate the predictive power of the reconstructed cellular morphology, a classification model was developed for identifying tumor cells. Each cell was labeled as a tumor cell if its nucleus centroid was located within a pathologist‐annotated tumor region; otherwise, it was considered a non‐tumor cell. The classification model was built using a four‐layer Residual Convolutional Neural Network (ResNet).

To provide a comprehensive comparison, the model on three distinct datasets were constructed and trained, each representing a different method for defining the cell's region: 1) Sliding Window Method: The first dataset was created using a sliding window approach, where small patches of 60 × 60 pixels were cropped starting from the top‐left corner of the image. This method represents a basic, grid‐based sampling of the tissue. 2) Nucleus‐Centered Method: The second dataset was generated by cropping a 60 × 60 pixel region centered on the location of each detected nucleus. This is a common and straightforward method for defining a cell's region in 2D analysis. 3) 3D‐Informed Method: The third and most novel dataset was constructed using the projected 2D area of the predicted 3D cellular regions (at the Z = 0 plane) as the input image. This method leverages the biomechanical constraints from the CellSqueeze3D framework.

Then, a comparative analysis was performed across the three models trained on these datasets. Their classification performance was evaluated using a suite of metrics, including Area Under the Curve (AUC), F1‐score, Precision, Recall, and Accuracy, to determine if the 3D‐informed cellular representation provided a more accurate and robust basis for cell‐type classification.

### Cell Morphology and Patient Prognosis

The relationship was explored between the reconstructed cell morphology, specifically the nucleus‐to‐cytoplasm (N/C) ratio, and patient prognosis. Inspired by a Nature study that found reduced size heterogeneity in metastatic cells, it was hypothesized that higher morphological heterogeneity (i.e., higher entropy of the N/C ratio distribution) might be linked to patient outcomes. The N/C ratio was correlated with patient survival data from the TCGA database. Kaplan‐Meier survival curves were constructed, and performed log‐rank tests to statistically assess the prognostic significance of the N/C ratio.

### N/C Ratio as a Predictor of Gene Mutations

The potential of the N/C ratio was investigated to serve as a morphological biomarker for predicting gene mutations. Mutation data was analyzed from various cancers in the TCGA database and correlated the N/C ratio with mutation status using fivefold cross‐validation. Mutations were identified that could be predicted with an AUROC greater than 0.65, and performed functional analysis of the associated genes to understand the biological relevance of these predictions.

### Statistical Analysis

All statistical analyses were performed using Python (version 3.9.1). The specific statistical methods applied in each experiment are detailed below:

### Data Pre‐Processing

Gene Expression Data: Raw counts were log2‐transformed after adding a pseudocount of 1, and then normalized using the transcripts per million (TPM) method.

Morphological Features (Cell Radius, N/C Ratio): Features were Z‐score normalized.

Survival Data: Overall survival (OS) was defined as the time from diagnosis to death. Patients alive at the last follow‐up were censored.

### Data Presentation

Continuous data are presented as mean ± standard deviation (SD) unless otherwise stated. Categorical data are presented as counts and percentages.

### Sample Size *(n)*

The sample size (n) for each experiment, which corresponds to the number of patients, image patches, or cells analyzed, is explicitly stated in the respective method subsections. No statistical method was used to predetermine sample size; all available samples meeting the quality control criteria from the TCGA database were included.

### Statistical Methods and Significance

Synthetic Validation: The two‐sided Kolmogorov‐Smirnov (KS) test was applied, a non‐parametric method, to evaluate the difference between the median N/C ratios from the optimized model and those from the random baseline across samples. The difference was found to be statistically significant (*p* < 0.05).

Correlation Analysis: The association between gene expression and the N/C ratio was assessed using Spearman's rank correlation coefficient, chosen for its robustness to non‐normal distributions. The resulting P values were adjusted for multiple hypothesis testing using the Benjamini‐Hochberg false discovery rate (FDR) procedure. An adjusted P value (FDR‐corrected q‐value) of less than 0.05 was considered statistically significant.

Survival Analysis: Patients were dichotomized into “High” and “Low” N/C ratio entropy groups based on the median value. Survival differences between groups were visualized with Kaplan‐Meier curves and compared using the log‐rank test (two‐sided). A *p* value < 0.05 indicated a statistically significant difference in survival.

Classification Performance: The performance of the cell‐type classifiers was evaluated using the Area Under the Receiver Operating Characteristic Curve (AUROC). Differences in AUC values between the 3D‐informed method and tow other methods were tested for statistical significance using *t*‐test (two‐sided).

## Conflict of Interest

The authors declare no conflict of interest.

## Author Contributions

Y.K. and H.L. conceived and designed the study. Y.K. was responsible for data analysis, visualization, and writing the original manuscript. H.L. contributed to the conceptualization, critical revision of the manuscript, and supervised the entire project.

## Supporting information



Supporting Information

Supporting Information

Supporting Information

## Data Availability

The data that support the findings of this study are available from the corresponding author upon reasonable request.
