# Supplementary material for: Biomechanics‐Driven 3D Architecture Inference from Histology Using CellSqueeze3D
Source: Adv Sci (Weinh). 2025 Dec 5;13(10):e18706. doi: 10.1002/advs.202518706 (PMC12915120; doi:10.1002/advs.202518706)
Supplement: Supplementary file 1 — Supporting Information [file ADVS-13-e18706-s002.docx]

Supporting Information

**CellSqueeze3D：Inferring 3D Cellular Architectures from Single Histology Sections via Biomechanical Constraint-Guided Optimization**

*Yan Kong and Hui Lu**


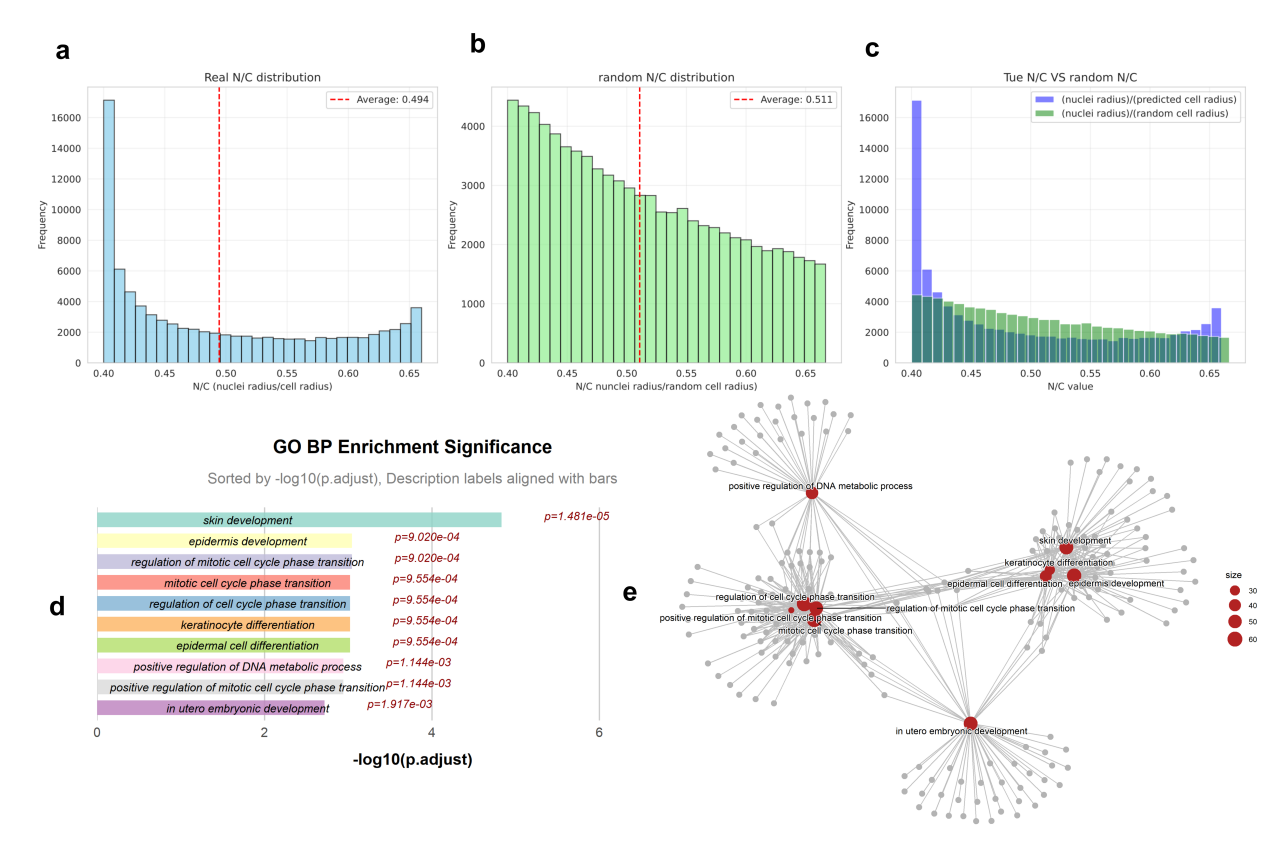
**Figure S1**. Model validation and functional analysis of nuclear-to-cytoplasmic (N/C) ratio features in the LUSC cohort (sample size, n=416; all patches are 1000 × 1000 pixels at 40X magnification). (a) Distribution of N/C ratios derived from the predicted cell radii. (b) Distribution of N/C ratios derived from randomly assigned cell radii. (c) Direct comparison of the two distributions shown in a and b. (d) Top 10 significantly enriched Gene Ontology Biological Process (GO BP) terms for genes correlated with N/C ratio entropy. (e) Network analysis illustrating the interconnections among the enriched GO BP terms from d.


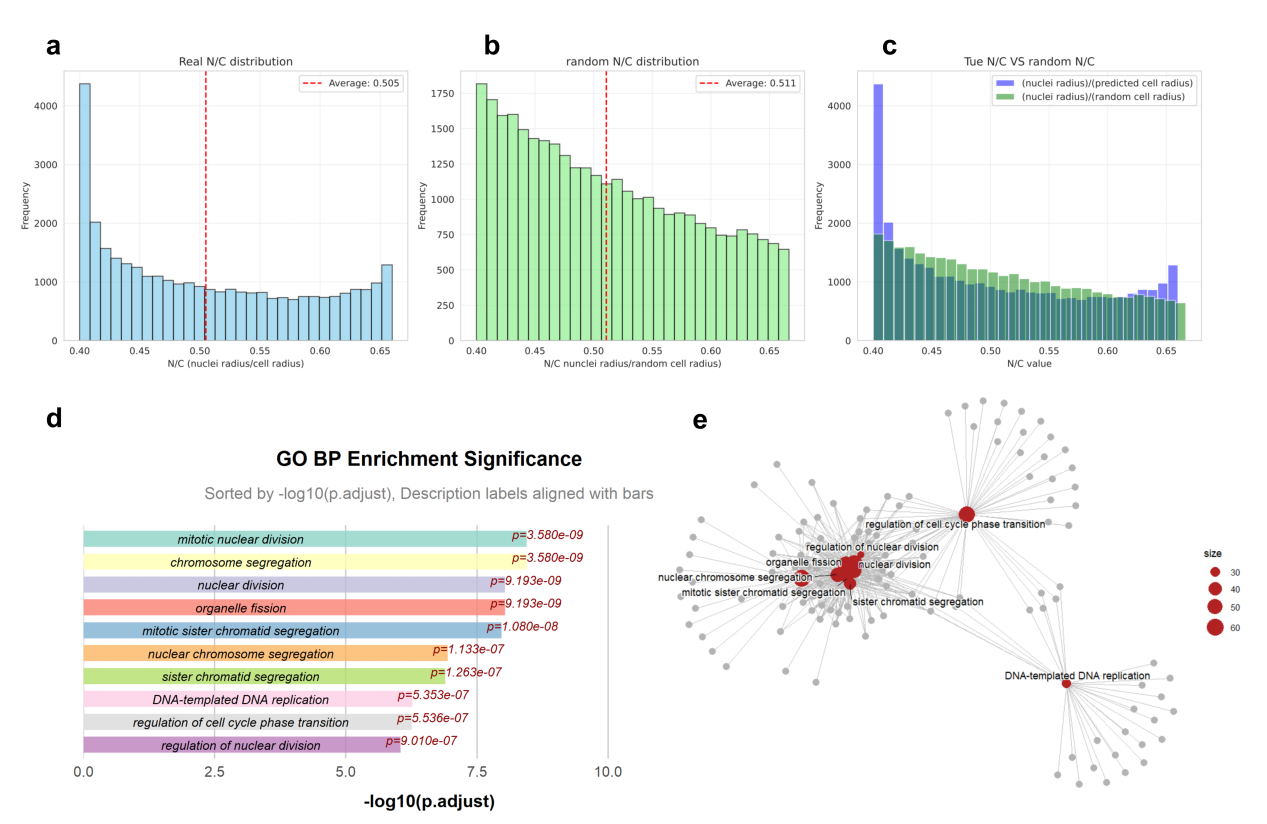

**Figure S2.** Model validation and functional analysis of nuclear-to-cytoplasmic (N/C) ratio features in the LGG cohort (sample size, n=212; all patches are 1000 × 1000 pixels at 40X magnification). (a) Distribution of N/C ratios derived from the predicted cell radii. (b) Distribution of N/C ratios derived from randomly assigned cell radii. (c) Direct comparison of the two distributions shown in a and b. (d) Top 10 significantly enriched Gene Ontology Biological Process (GO BP) terms for genes correlated with N/C ratio entropy. (e) Network analysis illustrating the interconnections among the enriched GO BP terms from d.


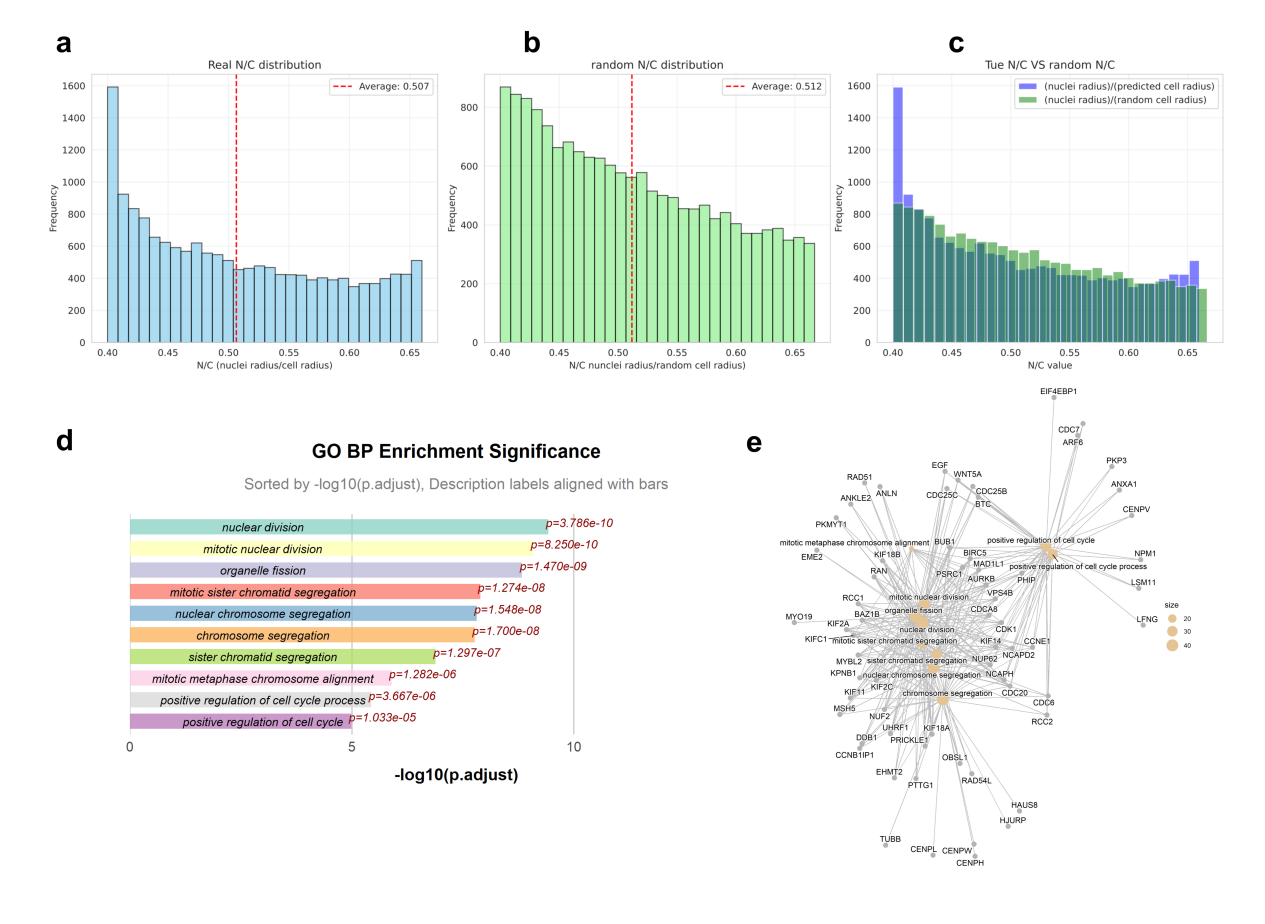


**Figure S3.** Comparison of predicted versus random cell radius distributions and functional analysis of genes associated with nuclear-to-cytoplasmic (N/C) ratios in the KICH cohort (sample size, n=116; all patches are 1000 × 1000 pixels at 40X magnification). (a) Distribution of N/C ratios derived from the predicted cell radii. (b) Distribution of N/C ratios derived from randomly assigned cell radii. (c) Direct comparison of the two distributions shown in a and b. (d) Top 10 significantly enriched Gene Ontology Biological Process (GO BP) terms for genes correlated with N/C ratio entropy. (e) Network analysis illustrating the interconnections among the enriched GO BP terms from d.

**Supplement Table 1.** Sample details for this study.
**Supplement Table 2.** Predicted entropy of nuclear-to-cytoplasmic (N/C) ratios and correlation analysis results.
